# Complement consumption in children with *Plasmodium falciparum *malaria

**DOI:** 10.1186/1475-2875-8-7

**Published:** 2009-01-09

**Authors:** Nancy K Nyakoe, Ronald P Taylor, Joseph N Makumi, John N Waitumbi

**Affiliations:** 1 Walter Reed Project, Kenya Medical Research Institute, Kisumu, Kenya; 2 Department of Biochemistry and Molecular Genetics, University of Virginia School of Medicine, Charlottesville, VA 22908, USA; 3 School of Pure and Applied Sciences, Kenyatta University, Nairobi, Kenya

## Abstract

**Background:**

Complement (C) can be activated during malaria, C components consumed and inflammatory mediators produced. This has potential to impair host innate defence.

**Methods:**

In a case-control study, C activation was assessed by measuring serum haemolytic activity (CH50), functional activity of each pathway and levels of C3a, C4a and C5a in children presenting at Kisumu District Hospital, western Kenya, with severe malarial anaemia (SMA) or uncomplicated malaria (UM).

**Results:**

CH50 median titers for lysis of sensitized sheep erythrocytes in SMA (8.6 U/mL) were below normal (34–70 U/mL) and were one-fourth the level in UM (34.6 U/mL (*P *< 0.001). Plasma C3a median levels were 10 times higher than in normals for

SMA (3,200 ng/ml) and for UM (3,500 ng/ml), indicating substantial C activation in both groups. Similar trends were obtained for C4a and C5a. The activities of all three C pathways were greatly reduced in SMA compared to UM (9.9% vs 83.4% for CP, 0.09% vs 30.7% for MBL and 36.8% vs 87.7% for AP respectively, *P *< 0.001).

**Conclusion:**

These results indicate that, while C activation occurs in both SMA and UM, C consumption is excessive in SMA. It is speculated that in SMA, consumption of C exceeds its regeneration.

## Background

There is ample evidence to indicate that malaria antigens, either on infected erythrocytes (iE), or as free antigens released from schizont rupture, or as immune complexes formed from antibodies that target the antigens, can all activate complement (C) [1-6]. Intravascular lysis of *Plasmodium falciparum *iE also releases breakdown products such as haemoglobin and haematin, which have inflammatory properties and can also activate C [7-9].

Low levels of C3, C4 and C1q in the sera of children with acute falciparum malaria have been demonstrated, thus implicating the classical pathway (CP). These studies have suggested that C activation may play a part in initiating changes that lead to the vascular damage seen in malaria [1]. The presence of cryoglobulins, circulating immune complexes (CIC) and hypocomplementaemia in patients with cerebral malaria has been reported, again implicating the CP. Wenisch *et al *reported activation of both the CP and alternative pathway (AP) that was marked by elevation of Bb, SC5b-9, and C4d [6]. Recent data has demonstrated C activation of the AP by moderate concentrations of haematin that promoted deposition of C3 activation and breakdown products on E, which may be indicative of a novel mechanism for the extravascular removal and lysis of E [9]. Moreover, mannose binding lectin (MBL), which binds to microbial surface carbohydrates [10], has been shown to bind malaria iE [2], likely causing activation of the MBL pathway. Other studies have shown that MBL-deficiency associated with MBL variant alleles that encode dysfunctional protein or decreased levels of the MBL can compromise the host's ability to fight malaria [11,12].

C activation also leads to generation of very potent pro-inflammatory mediators, especially C5a and C3a [13-15]. C3a and C5a are also chemo-attractant agents and are involved in recruitment of inflammatory cells and although they normally play roles in immunological defence, they may promote tissue injury and destruction of innocent bystander cells. Protection against C-mediated damage is provided by cell-associated C regulatory proteins such as CR1, CD55 and CD59, which are expressed on E and other cells exposed to the bloodstream [16]. These proteins inhibit C activation, and in addition, E can remove CIC from the bloodstream via CR1 [17,18]. However, the removal of CIC by E can promote substantial loss of CR1 [18-21]. Indeed, in children with malaria, both E-associated CR1 and CD55 are substantially reduced, and therefore the E may be more susceptible to C-mediated damage [22-25]. Excessive C activation in malaria could also deplete serum C and the ensuing hypocomplementaemia would reduce the ability of the host to fight bacterial infections. In fact, bacteraemia is a common complication in malaria [26], but its association with hypocomplementaemia has not been ascertained.

In this study, children presenting at Kisumu District Hospital, western Kenya with either severe malarial anaemia (SMA) or with uncomplicated malaria (UM) were enrolled. Their C status was examined by measuring serum haemolytic activity, functional activity of the three arms of the C cascade, and levels of C split products (C3a, C4a, C5a).

## Methods

### Study site and design

The study was a hospital-based prospective case-control. 60 patients with severe malaria (SMA) were matched by age (± 2 months) and sex to children with uncomplicated malaria (UM). Both groups were recruited from the paediatric ward of Kisumu District Hospital, western Kenya. Specimens for research were transported to the WRP/KEMRI research laboratory in Kondele, Kisumu where the assays were performed.

### Sample size calculation and statistics

The sample size of 60 in each arm was based on a previous similar study that recruited 30 subjects in each arm and showed a statistically significant difference in CR1 levels in children with complicated malaria and those without the disease [25]. Based on the hypothesis that complement utilization may be more complex than removal of CR1, the sample size was doubled. Statistical analysis was carried out using Graph pad prism version 5 (GraphPad, San Diego CA). Wilcoxon matched-pairs signed-ranks test was used to determine if the two cohorts have different medians. The effectiveness of matching was determined using nonparametric Spearman correlation test.

### Ethical consideration

Participation into the study was under Approved Protocol # 1145 obtained from the Ethical Review Committee of the Kenya Medical Research Institute, Nairobi and the Walter Reed Army Research Institute of Human Use Research Committee, Silver Spring, Maryland, USA.

### Study population

Two groups of children were enrolled. Group one (SMA) comprized children (age ≤ 5 yrs) admitted to the participating hospital with asexual *P*. *falciparum *parasitaemia confirmed by a positive Giemsa-stained blood smear and anaemia (haemoglobin ≤ 6 g/dL). Group two (UM) were age (± 2 months) and sex matched children with symptomatic uncomplicated malaria. Inclusion and exclusion criteria were as previously published [23,25]. Thin and thick blood film preparation and staining for microscopic diagnosis of malaria was as described [27].

### Serum samples

Blood samples were collected into serum separation tubes devoid of clot activator and allowed to remain at room temperature for 30 minutes. Serum was then separated from the clot by centrifugation at 2,000 × *g *for 5 min at 2–4°C. The serum was aspirated, aliquoted and kept at -70°C until examined. For use, samples were thawed at room temperature and then kept on ice until they were analysed.

### Determination of haemolytic complement titer (CH50)

Complement titers, based on the classical haemolytic assay for lysis of antibody-sensitized sheep E, were performed as described previously [28,29]. Briefly, 2 × 10^8 ^E/ml were opsonized with rabbit anti-sheep haemolysin (Sigma-Aldrich, MO) by adding an equal volume of a 1:160 dilution of haemolysin to the E suspension and the mixture was then incubated at 37°C in a shaking water bath for 30 minutes. The sensitized E were then centrifuged at 3,000 rpm for five minutes and washed three times with gelatin veronal buffer (GVB) (Sigma-Aldrich Inc, supplemented with 10 mM EDTA (GVBE). Sensitized E were stored in GVBE supplemented with 2.5% glucose, 0.03% NaN_3 _at 4°C for not more than two weeks. For use, sensitized erythrocytes were washed with GVB containing Mg^2+ ^and Ca^2+ ^(GVB++).

Patient sera (in duplicates) were serially diluted in a 96 micro-well plate in GVB++ to give a final dilutions of 1/320. To each serum dilution, 25 μl of 5% sensitized E was added followed by incubation at 37°C for 60 min. To stop the reaction, 100 μl of GVBE was added to all wells. Control wells included spontaneous lysis of opsonized E without serum and 100% lysis well in which complete lysis was achieved by adding 1% triton X. The plate was centrifuged at 3,000 rpm in a plate centrifuge (Marathon 3000R, Fisher Scientific, MA) at 4°C for 5 min to pellet un-lysed cells. 100 μl of supernatant was then transferred to a flat-bottomed plate and the absorbance readings of released hemoglobin read at 415 nm (Vmax Kinetic micro plate reader, Molecular Devices, USA). The degree of lysis (y) was determined from the formula: y = (sample OD – spontaneous lysis control OD)/(OD of 100% lysis – spontaneous lysis control OD). A standard curve was generated from the serial dilutions of each sample and used to determine the serum titer that causes 50% haemolysis of sensitized sheep E.

### Determination of functional complement activity in the pathways

The functional C activity in each pathway was evaluated using ELISA kits (Wieslab, Euro-Diagnostica, Malmo, Sweden), that are supplied pre-coated with specific activators for the CP, MBL and AP. Serum samples were diluted into buffers containing specific blockers to ensure that only the respective pathway was activated. For CP and MBL, samples were diluted at 1:100, and 1:18 for the AP. For quality control of the assay and for calculation of the functional activities, positive and negative controls with known activities were used as recommended by the kit manufacturer. Briefly, 100 μL of each diluted sample and control were added in duplicate to the wells of the respective pathway-specific plates and incubated for 70 min at 37°C. Wells were then aspirated and washed thrice with wash buffer. Then 100 μL of conjugate containing alkaline phosphatase-labeled antibodies to C5b-9 was added to each well and incubated for 30 min at 25°C. Wells were again washed thrice and 100 μL of substrate [(3,3',5,5' tetramethylbenzidine (TMB)] added and the samples were incubated for 30 min at 25°C to allow for color development. Absorbance was then immediately read at 405 nm. Functional activity for the respective pathways was then calculated using the OD values of the positive and negative controls.

### Detection of complement activation fragments

Plasma levels of C fragments were measured using commercial ELISA kits (BD, Biosciences Pharmigen, San Diego, CA) that detect C3a-desArg, C4a-desArg and C5a-desArg, the stable metabolites of C3a, C4a and C5a. In this assay, the plates are supplied pre-coated with monoclonal antibodies that are respectively specific for human C3a-desArg, C4a-desArg and C5a-desArg. Plasma samples for C3a-desArg and C4a-desArg were diluted at 1:500 and 1:5 for C5a-dersArg. To allow quantification of the complement fragments, a calibration curve was made with plasma (supplied with kits) containing known amounts of each fragment. Then 100 μl of each diluted sample and standard were added in duplicate wells and incubated for two hours at room temperature. Wells were then washed four times with wash buffer provided in the kits. Working detector was prepared by mixing biotinylated polyclonal anti-human C3a, or C4a and or C5a antibody and streptavidin-horseradish peroxidase. Then 100 μl of working detector was added to each well, the plate was sealed and incubated at room temperature for one hour. Wells were again washed six times and then 100 μl of TMB substrate added and the plates were incubated for 30 min at room temperature. The reaction was stopped with 50 μl of 1 M phosphoric acid and the absorbance was then read at 450 nm. The concentration of respective fragments in each sample was extrapolated from the standard curves.

### PCR detection of C4AQ0 and C4BQ0 null alleles

Details of the touch down PCR for detection of C4AQ0 and C4BQ0 null alleles have been described previously [30].

Briefly, four oligonucleotide primer pairs specific for C4A isotypes and C4B isotypes were used for amplification. The primers pairs Aup (5'-gCatgCtCCtgtCtaaCaCtggaC-3') and L3 (5'-gCggatCCagCagtttCggaag-3') were used to amplify a 377 bp fragment of isotype A while Bup (5'-tgCtCCtatgtatCaCtggagaga-3') and L3 amplify the same size fragment of isotype B. Adown (5'-aggaCCCCtgtCCagtgttagaC-3') and L4 (5'-ataggatCCtaaggtCCCtgggCCt-3') were used to amplify a 578 bp fragment of isotype A while Bdown (aggaCCtCtCtCCagtgatagat) and L4 amplify same size fragment of isotype B. Absence of any of these fragments indicate lack of that allele. PCR was carried out in a 25 μl reaction volume containing 1 μM of each of the above primers, 1× gene amplification PCR buffer, 1.5 mM MgCl_2_, 200 μM dNTPs and 2 U of Taq polymerase. Cycling was performed on a DNA engine (Tetrad PTC-225, MJ Research Inc. MA), with an initial denaturation step at 94°C for 5 min, followed by 12 cycles at 94°C for 30 s, decreasing annealing temperature by 1°C steps for each two cycles from 68°C to 63°C for 1 min and extension temperature at 72°C for 1 min. The remaining 28 cycles were run with annealing temperature of 63°C and the same denaturation and extension conditions. Following amplification 15 μl of PCR amplicons were analysed by gel electrophoresis on 2% agarose (Sigma-Aldrich Inc).

## Results

### Subjects enrolled

Although the study enrolled 60 cases and 60 controls whose characteristics are shown in Table 1, not all samples were available for all the analyses. The median age for both groups was 16.5 months. Parasite median densities and interquartile range in the SMA group was 58,000 iE/mL (12,200–133,000) and were not statistically different from that of UM control group, 39,000 iE/mL (6,400–105,000), even after log transformation. The mean Hb level in cases was 4.5 ± 1.0 g/dL and 8.9 ± 1.3 g/dL in the controls.

**Table 1:** Demographics and clinical characteristics of patients enrolled in the malarial anaemia case control study.

**Characteristics**	**Severe malaria**	**Uncomplicated malaria**	**P**
Sample size (N)	60	60	NC
Median age in months and (interquartile range)	16.5 (10–21)	16.5 (9–23)	NC
Median parasitaemia (iE/μl) (interquartile range)	58,000 (12,200–133,000)	39,000 (6400–105,000)	0.66
Hb level (g/dl)	4.5 ± 1.0	8.9 ± 1.3	NC

Note: P = Wilcoxon matched-pairs signed-ranks test. NC = not necessary.

### Serum of children with SMA have reduced haemolytic activity

**Figure 1:**
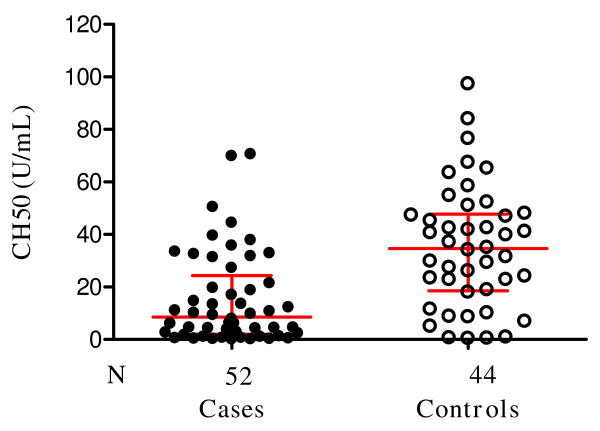
**Scatter plot showing the median and interquartile C hemolytic titer in children with severe malaria anemia (cases) and those with uncomplicated malaria (controls)**. The CH50 in cases was 8.6 U/mL (2.1 to 24.4) compared to 34.6 U/mL (18.5 to 47.7) in the controls (*P *< 0.001). Wilcoxon matched-pairs signed-ranks test was performed with the 44 paired samples.

As shown in Figure 1, samples from 52 SMA and 44 UM were available for analysis. Children with SMA had very low C activity compared to children with UM. The CH50 median levels and interquartile range in the SMA group was 8.6 U/mL (2.1 to 24.4) (normal = 34–70 U/mL) compared to 34.6 U/mL (18.5 to 47.7) in the UM group (*P *< 0.001), with 50% of the children in the SMA group having no detectable complement activity. Wilcoxon matched-pairs signed-ranks test was performed with the 44 paired samples.

### Levels of the three complement pathways are consumed in children with severe malarial anaemia

**Figure 2:**
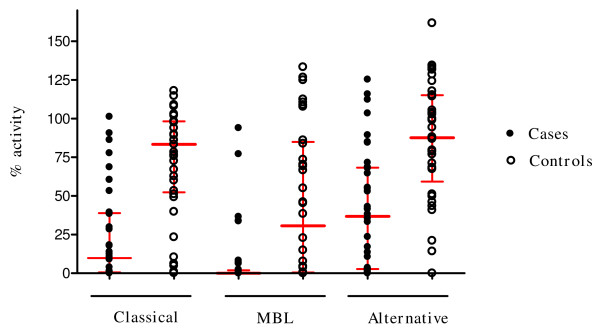
**Aligned dot plot showing median and interquartile % functional activity in each complement pathway in children with severe malaria anaemia (cases) and those with uncomplicated malaria (controls)**. The median functional activity in cases was significantly reduced compared to the controls (9.9% vs 83.4% for CP, 0.09% vs 30.7 for MBL and 36.8% vs 87.7% for AP respectively, *P *< 0.001). Wilcoxon matched-pairs signed-ranks test was performed with the 36 paired samples.

Figure 2 shows the median and interquartile % functional C activity in each arm of the cascade in the SMA and UM groups. In each pathway, the functional activity was greatly reduced in cases compared to the controls (median = 9.9% vs 83.4% for CP, 0.09% vs 30.7% for MBL and 36.8% vs 87.7% for AP respectively, *P *< 0.001. Moreover, in the SMA group, more than half of the children had no measurable activity in all three C pathways.

### Cases and controls have elevated levels of the anaphylatoxins C4a, C3a and C5a

**Figure 3:**
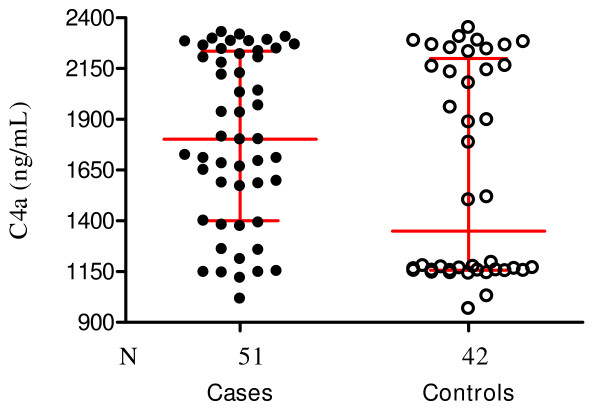
**Scatter plot showing the median and interquartile C4a levels in children with severe malaria anaemia (cases) and those with uncomplicated malaria (controls)**. The median C4a levels in cases was 1,800 ng/mL (1,400–2,200) and 1,350 ng/ml (1,160–2,200) in the controls (*P *= 0.08). Wilcoxon matched-pairs signed-ranks test was performed with the 42 paired samples.

**Figure 4:**
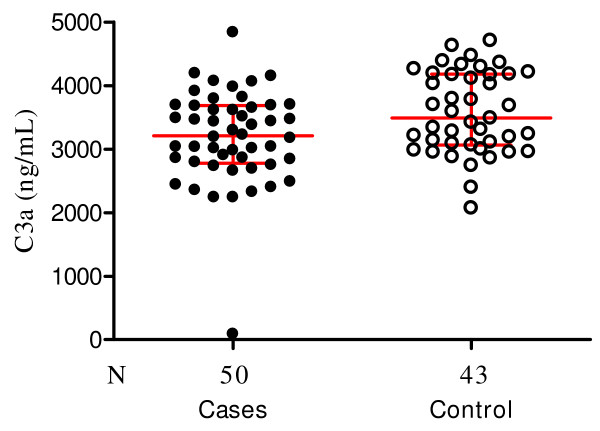
**Scatter plot showing the median and interquartile C3a levels in children with severe malaria anaemia (cases) and those with uncomplicated malaria (controls)**. The median values were significantly higher in the controls (3,500 ng/ml, 3,100–4,200) compared to the cases (3,200 ng/mL, 2,800–3,700) *P *= 0.02). Wilcoxon matched-pairs signed-ranks test was performed with the 43 paired samples.

**Figure 5:**
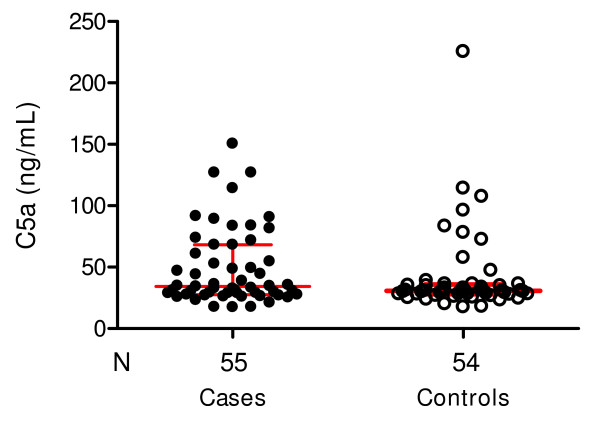
**Scatter plots showing median and interquartile range of C5a levels in severe malarial anaemia (cases) and those with uncomplicated malaria (controls)**. The median level in the cases (34.3 ng/mL, 27.7–68.2) was comparable to the level in the control group (30.7 ng/mL, 28.1–35.8), *P *= 0.35). Wilcoxon matched-pairs signed-ranks test was performed with the 54 paired samples.

Plasma anaphylatoxin levels for C4a, C3a and C5a which are good indicators of the extent of C activation were markedly elevated in both the SMA and UM groups (Figures 3, 4 and 5). The C4a levels, which reflect upstream activation of the CP and MBL pathways are shown in Figure 3. The median and interquartile C4a levels in both cases and controls were markedly elevated in SMA (1,800 ng/mL, 1,400–2,200) and UM (1,350 ng/mL, 1,160–2,200) but there was no significant difference between the two groups (*P *= 0.08). The normal plasma levels of C4a range between 63 and 235 ng/mL. Wilcoxon matched-pairs signed-ranks test was performed with the 42 paired samples.

Levels of C3a, which serve as indices of activation of all three C pathways, showed marked elevations (Figure 4) in both SMA and UM groups, but in contrast with the findings for C4a, the median values and interquartile range were slightly higher for UM (3,500 ng/mL, 3,100–4,200) compared to SMA (3200 ng/ml, 2,800–3,700 (*P *= 0.02). Normal plasma C3a levels range between 250–700 ng/mL. Wilcoxon matched-pairs signed-ranks test was performed with the 43 paired samples.

The median levels of C5a (Figure 5), which represent downstream activation of C and are indicative of initiation of assembly of the membrane attack complex (MAC), were markedly elevated in both groups (SMA = (34.3 ng/mL, UM = 30.7 ng/mL, *P *= 0.35) compared to normal levels (1.3–8 ng/mL), thus suggesting that some intravascular haemolysis may occur in both groups. Wilcoxon matched-pairs signed-ranks test was performed with the 54 paired samples.

### Genotyping of C4 null alleles

The 378 and 578 bp C4A isotypes were present in all the samples tested. Five individuals were found to have the C4B null alleles and four of these were in the uncomplicated malaria group, thus uncoupling this genetic factor from the low C levels.

## Discussion

All three pathways of C activation have been variously implicated as activation targets in malaria [1-4,6]. The study reported here supports these observations and further demonstrate the extent to which each pathway of C is consumed and therefore potentially unavailable for the host defence. The results indicate that while C activation occurs in children with mild and severe malaria, in SMA, C consumption is more severe. Indeed, in the SMA group, the median CH50, a measure of total C activity was less than half that in control group, with 50% of the children having no detectable complement activity in any of the pathways (Figures 1 and 2), clearly indicating substantial consumption of C in all three pathways in children with SMA. Since hypocomplementaemia compromizes defence against bacteria infection, this finding may partly explain why bacteraemia is a common complication in malaria [26,31].

Depletion of the CP may be linked to the high levels of CIC that have been reported in children with SMA [1,32,33]. In the MBL pathway, it is the binding of MBL to mannose and N-acetyl glucosamine residues of microorganisms that activates complement. It is not clear whether malarial antigen carbohydrate moieties activate MBL, but proteins on the surface of malaria iE are recognized by MBL [2,34]. Studies are ongoing to determine whether the observed low levels of MBL in children with SMA are related to variant MBL alleles that have been associated with increased susceptibility to severe malaria [11,12].

Activation of C generates potent inflammatory peptides, especially C5a and C3a, and these agents have a broad spectrum of biological functions including generation of cytokines, such as TNF [13-15]. In vivo, the normally short lived C3a, C4a and C5a are cleaved to more stable desArg metabolites whose measurements can be used to draw reliable conclusions about ongoing C activation activities [35]. Contrary to the expectation that increased C consumption as seen in the SMA group (Figures 1 and 2) would result in significantly higher C activation products, the levels of C3a-desArg were in fact higher in the UM group, and similarly elevated for C4a-desArg and C5a-desArg in the two groups (Figure 3). It is unclear why this is so, but speculations are that, in children with SMA, consumption of C exceeds its regeneration.

In this study, the median plasma C5a-desArg levels were markedly elevated in both groups (cases = 34.3 ng/mL, controls = 30.7 ng/mL, Figure 5), compared to normal levels (ranges from 1.3–8 ng/mL). Elevated C5a may indicate significant intravascular haemolysis due to assembly of MAC. However, because levels of C5a in SMA were not significantly different from those in UM, these results suggest that the excessive destruction of E that occurs in children with severe malarial anaemia may not be due to assembly of the MAC. Previous data implicate complement-mediated extra-vascular destruction of red blood cells [23-25]. Moreover, although the AP was not depleted as much as the other two pathways, it was reduced considerably, and in fact more than half of the children with SM had no AP activity (Figure 2). This could reflect C activation mediated by haematin released by intravascular haemolysis. As noted, this reaction can deposit C3 activation products on young E that express high levels of CR1, possibly leading to their early extravascular clearance and destruction [9].

Although rare, complement deficiency can also be caused by genetic factors [36-38]. Of these genetic deficiencies, the most common involve C4 [39]. Carriers of complement deficiencies suffer from immune complex related diseases such as systemic lupus erythematosus [19,21] and have an increased vulnerability to microbial infection [40]. However, examination of C4 null alleles suggested that the low C activity observed in patients with SMA are not related to genetic defect of C4 genes.

## Conclusion

In conclusion, the findings in this study indicate substantial activation of C irrespective of malarial clinical status. However, the high level of C consumption observed in children with SMA compared to UM indicate that in the SMA, the consumption remains uncompensated. The actual malarial components that can activate C have not been identified, but are thought to include malarial antigens on the surface of iRBC, antigens that are released during schizont rapture either as free antigens or bound to antibodies, and free haematin [1,2,9,32,34]. The cross sectional nature of the current study makes it difficulty to draw definite conclusions regarding causal relationship between C utilization and susceptibility to severe malaria. It could for example be argued that the SMA group represent those children with prolonged symptoms before seeking medical treatments while UM group represent those that are recovering or those who have had a short duration of illness. It will be possible to address such issues in future studies that will have a longitudinal design.

## Competing interests

The authors declare that they have no competing interests.

## Authors' contributions

NKN conducted all the experiments, helped in data analysis and edited the manuscript. RPT assisted in study design and edited the manuscript. JNM assisted in study design and data interpretaion. JNW designed the study, directed the work and drafted the manuscript. All authors have read and approved the final manuscript.

## Disclaimer

The opinions or assertions contained herein are the private views of the authors, and are not to be construed as official, or as reflecting true views of the Department of the Army or the Department of Defense.

## References

[B1] Adam C, Geniteau M, Gougerot-Pocidalo M, Verroust P, Lebras J, Gibert C, Morel-Maroger L (1981). Cryoglobulins, circulating immune complexes, and complement activation in cerebral malaria. Infect Immun.

[B2] Garred P, Nielsen MA, Kurtzhals JAL, Malhotra R, Madsen HO, Goka BQ, Akanmori BD, Sim RB, Hviid L (2003). Mannose-binding lectin is a disease modifier in clinical malaria and may function as opsonin for *Plasmodium falciparum*-infected erythrocytes[erratum appears in Infect Immun. 2003 71:6687]. Infect Immun.

[B3] Glew RH, Atkinson JP, Frank MM, Collins WE, Neva FA (1975). Serum complement and immunity in experimental simian malaria. I. Cyclical alterations in C4 related to schizont rupture. J Infect Dis.

[B4] Greenwood BM, Brueton MJ (1974). Complement activation in children with acute malaria. Clin Exp Immunol.

[B5] Houba V, Williams AI (1972). Soluble serum antigens of *P. falciparum *in Nigerians. II. Immunochemical studies. Afr J Med Sci.

[B6] Wenisch C, Spitzauer S, Florris-Linau K, Rumpold H, Vannaphan S, Parschalk B, Graninger W, Looareesuwan S (1997). Complement activation in severe *Plasmodium falciparum *malaria. Clin Immunol Immunopathol.

[B7] Kaca W, Roth R (1995). Activation of complement by human hemoglobin and by mixtures of hemoglobin and bacterial endotoxin. Biochim Biophys Acta.

[B8] Omodeo-Sale F, Motti A, Dondorp A, White NJ, Taramelli D (2005). Destabilisation and subsequent lysis of human erythrocytes induced by *Plasmodium falciparum *haem products. Eur J Haematol.

[B9] Pawluczkowycz AW, Lindorfer MA, Waitumbi JN, Taylor RP (2007). Hematin promotes complement alternative pathway-mediated deposition of C3 activation fragments on human erythrocytes: potential implications for the pathogenesis of anemia in malaria. J Immunol.

[B10] Roozendaal R, Carroll MC (2006). Emerging patterns in complement-mediated pathogen recognition[comment]. Cell.

[B11] Boldt ABW, Luty A, Grobusch MP, Dietz K, Dzeing A, Kombila M, Kremsner PG, Kun JFJ (2006). Association of a new mannose-binding lectin variant with severe malaria in Gabonese children. Genes Immun.

[B12] Luty AJ, Kun JF, Kremsner PG (1998). Mannose-binding lectin plasma levels and gene polymorphisms in *Plasmodium falciparum *malaria. J Infect Dis.

[B13] Chen N-J, Mirtsos C, Suh D, Lu Y-C, Lin W-J, McKerlie C, Lee T, Baribault H, Tian H, Yeh W-C (2007). C5L2 is critical for the biological activities of the anaphylatoxins C5a and C3a. Nature.

[B14] Huber-Lang M, Sarma JV, Zetoune FS, Rittirsch D, Neff TA, McGuire SR, Lambris JD, Warner RL, Flierl MA, Hoesel LM (2006). Generation of C5a in the absence of C3: a new complement activation pathway. Nat Med.

[B15] Skokowa J, Ali SR, Felda O, Kumar V, Konrad S, Shushakova N, Schmidt RE, Piekorz RP, Nurnberg B, Spicher K (2005). Macrophages induce the inflammatory response in the pulmonary Arthus reaction through G alpha i2 activation that controls C5aR and Fc receptor cooperation. J Immunol.

[B16] Morgan BP, Harris CL (1999). Complement Regulatory Proteins.

[B17] Ahearn JM, Fearon DT (1989). Structure and function of the complement receptors, CR1 (CD35) and CR2 (CD21). Adv Immunol.

[B18] Nardin A, Lindorfer MA, Taylor RP (1999). How are immune complexes bound to the primate erythrocyte complement receptor transferred to acceptor phagocytic cells?. Mol Immunol.

[B19] Manderson AP, Botto M, Walport MJ (2004). The role of complement in the development of systemic lupus erythematosus. Annu Rev Immunol.

[B20] Reinagel ML, Gezen M, Ferguson PJ, Kuhn S, Martin EN, Taylor RP (1997). The primate erythrocyte complement receptor (CR1) as a privileged site: binding of immunoglobulin G to erythrocyte CR1 does not target erythrocytes for phagocytosis. Blood.

[B21] Walport MJ, Davies KA (1996). Complement and immune complexes. Res Immunol.

[B22] Craig ML, Waitumbi JN, Taylor RP (2005). Processing of C3b-opsonized immune complexes bound to non-complement receptor 1 (CR1) sites on red cells: phagocytosis, transfer, and associations with CR1. J Immunol.

[B23] Stoute JA, Odindo AO, Owuor BO, Mibei EK, Opollo MO, Waitumbi JN (2003). Loss of red blood cell-complement regulatory proteins and increased levels of circulating immune complexes are associated with severe malarial anemia. J Infect Dis.

[B24] Waitumbi JN, Donvito B, Kisserli A, Cohen JHM, Stoute JA (2004). Age-related changes in red blood cell complement regulatory proteins and susceptibility to severe malaria. J Infect Dis.

[B25] Waitumbi JN, Opollo MO, Muga RO, Misore AO, Stoute JA (2000). Red cell surface changes and erythrophagocytosis in children with severe *Plasmodium falciparum *anemia. Blood.

[B26] Berkley J, Mwarumba S, Bramham K, Lowe B, Marsh K (1999). Bacteraemia complicating severe malaria in children. Trans R Soc Trop Med Hyg.

[B27] Ohrt C, O'Meara WP, Remich S, McEvoy P, Ogutu B, Mtalib R, Odera JS (2008). Pilot assessment of the sensitivity of the malaria thin film. Malar J.

[B28] DiLillo DJ, Pawluczkowycz AW, Peng W, Kennedy AD, Beum PV, Lindorfer MA, Taylor RP (2006). Selective and efficient inhibition of the alternative pathway of complement by a mAb that recognizes C3b/iC3b. Mol Immunol.

[B29] Kennedy AD, Solga MD, Schuman TA, Chi AW, Lindorfer MA, Sutherland WM, Foley PL, Taylor RP (2003). An anti-C3b(i) mAb enhances complement activation, C3b(i) deposition, and killing of CD20+ cells by rituximab. Blood.

[B30] Barba GM, Braun-Heimer L, Rittner C, Schneider PM (1994). A new PCR-based typing of the Rodgers and Chido antigenic determinants of the fourth component of human complement. Eur J Immunogenet.

[B31] Bronzan RN, Taylor TE, Mwenechanya J, Tembo M, Kayira K, Bwanaisa L, Njobvu A, Kondowe W, Chalira C, Walsh AL, Phiri A, Wilson LK, Molyneux ME, Graham SM (2007). Bacteremia in Malawian children with severe malaria: prevalence, etiology, HIV coinfection, and outcome. J Infect Dis.

[B32] June CH, Contreras CE, Perrin LH, Lambert PH, Miescher PA (1979). Circulating and tissue-bound immune complex formation in murine malaria. J Immunol.

[B33] Mibei EK, Orago ASS, Stoute JA (2005). Immune complex levels in children with severe *Plasmodium falciparum *malaria. Am J Trop Med Hyg.

[B34] Klabunde J, Uhlemann A-C, Tebo AE, Kimmel J, Schwarz RT, Kremsner PG, Kun JFJ (2002). Recognition of *Plasmodium falciparum *proteins by mannan-binding lectin, a component of the human innate immune system. Parasitol Res.

[B35] Bokisch VA, Muller-Eberhard HJ (1970). Anaphylatoxin inactivator of human plasma: its isolation and characterization as a carboxypeptidase. J Clin Invest.

[B36] Agrawal R (2006). Complement Deficiency. WebMD>eMedicine Specialties.

[B37] Frank MM (2000). Complement deficiencies. Pediatr Clin North Am.

[B38] Nusinow SR, Zuraw BL, Curd JG (1985). The hereditary and acquired deficiencies of complement. Med Clin North Am.

[B39] Hauptmann G, Tappeiner G, Schifferli JA (1988). Inherited deficiency of the fourth component of human complement. Immunodefic Rev.

[B40] Seelen MA, Roos A, Wieslander J, Mollnes TE, Sjoholm AG, Wurzner R, Loos M, Tedesco F, Sim RB, Garred P (2005). Functional analysis of the classical, alternative, and MBL pathways of the complement system: standardization and validation of a simple ELISA. J Immunol Methods.

